# Propylene Glycol Toxicity in Adolescent with Refractory Myoclonic Status Epilepticus

**DOI:** 10.1155/2017/2979486

**Published:** 2017-02-26

**Authors:** Kara A. Bjur, Bryan C. Cannon, Anthony L. Fine, Matthew J. Ritter, Kerry E. Schueler, Michael E. Nemergut

**Affiliations:** Mayo Clinic, Rochester, MN, USA

## Abstract

Propylene glycol (PG) is a solvent commonly used in medications that, while benign at low doses, may cause toxicity in adults and children at high doses. We describe a case and the physiologic sequelae of propylene glycol toxicity manifested in a critically ill adolescent male with refractory myoclonic status epilepticus aggressively treated with multiple PG-containing medications (lorazepam, phenobarbital, and pentobarbital)—all within accepted dosing guidelines and a total daily PG exposure previously recognized to be safe. Hemodynamic measurements by bedside echocardiography during clinical toxicity are also reported. Clinicians should have a high index of suspicion for propylene glycol toxicity in patients treated with PG-containing medications even when the total PG exposure is lower than currently accepted limits.

## 1. Introduction

Propylene glycol (PG) is an excipient commonly used in medications and is “generally recognized as safe” by the US Food and Drug Administration under 21 CFR §184.1666 [1]. Clinical toxicity has been well described in both adults and children receiving PG-containing medications including lorazepam, diazepam, pentobarbital, trimethoprim-sulfamethoxazole, esmolol, phenytoin, phenobarbital, etomidate, nitroglycerin, multivitamin preparations, and silver sulfadiazine [2]. A typical presentation for PG toxicity is the appearance of an anion and osmol gap metabolic acidosis associated with hemodynamic lability, renal insufficiency, and, if untreated, multiorgan system dysfunction. Fundamental to the appearance of this toxidrome is the provision of a “toxic” dose of PG as numerous therapeutic drugs commonly used in the intensive care unit contain PG, and low doses are believed to be safe. What is considered toxic is currently unknown. While the World Health Organization recommends a maximum ingestion of PG in food additives of 25 mg/kg/day, this limit does not apply to drug excipients where toxicity is reported at much higher dosages [2–4]. There are no formal recommendations regarding daily maximum PG doses in the United States. Using the recommended maximum adult lorazepam dose (166 mg/day), 69 g/day of PG is presumed safe in a 70 kg adult with normal renal and hepatic function [2]. When extrapolated to the pediatric population (daily maximum lorazepam dose 2.4 mg/kg or 0.1 mg/kg/hour), approximately 1 g/kg/day would be the upper limit of PG exposure. Maximum daily pediatric doses of other commonly used intravenous medications corresponding to this limit have been proposed to avoid PG toxicity in children [5]; however, evidence in support of these limits are weak and previous reports exist of children receiving much higher doses of PG-containing medications (9 g/kg/day) without clinical toxicity [6]. Use of PG-containing medications is exceedingly common, but the presence of a proposed dosing limit in children combined with numerous reports in the pediatric literature exceeding this limit without the development of PG toxicity has made prescribing limits for safe dosing of PG a clinical conundrum for all practitioners who care for critically ill children. We present a case of PG toxicity and associated physiologic sequelae of an adolescent male, unique in that he received PG at doses lower than the prescribed limit and previously thought to be safe.

## 2. Case Presentation

**Figure 1:**
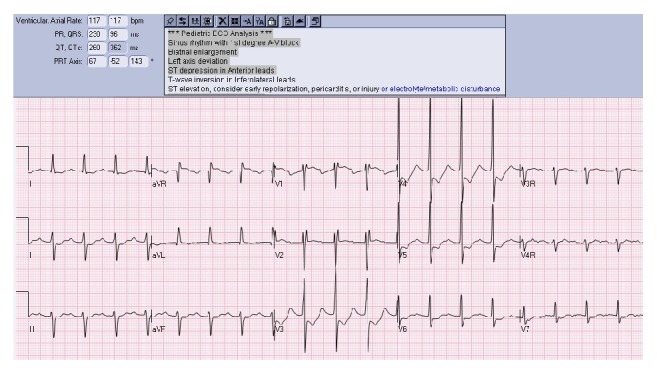
Electrocardiogram.

A 13-year-old 32 kg male with a past medical history significant for dystonia and learning difficulties was directly admitted to the pediatric intensive care unit for evaluation and treatment of new-onset myoclonic status epilepticus. Burst suppression was achieved on hospital day one following administration and titration of the following medications (total daily mg/kg): lorazepam (0.4 mg/kg), levetiracetam (50 mg/kg), fosphenytoin (30 PE/kg), phenobarbital (40 mg/kg), midazolam (2 mg/kg/hr), pentobarbital (5 mg/kg load, 4 mg/kg/hr), pyridoxine (100 mg), and isoflurane (0.5%). Burst suppression was maintained for the subsequent two days with midazolam (2 mg/kg/hr), pentobarbital (4 mg/kg/hr), and isoflurane (titrated to burst suppression). The average daily PG exposure during the first three hospital days was 1 g/kg/day, 0.8 g/kg/day, and 0.8 g/kg/day, respectively. Renal and hepatic function during the first three days was within normal limits. On hospital day three, the patient developed acute, severe distributive shock refractory to four separate vasopressors at high doses (maximum infusion rate): norepinephrine (0.4 mcg/kg/min), epinephrine (0.3 mcg/kg/min), dopamine (20 mcg/kg/min), and vasopressin (0.15 units/kg/hr). In addition, the vasoplegia was refractory to administration of methylene blue. Physical examination was pertinent for warm extremities, flash capillary refill, bounding pulses, and a hyperdynamic precordium. Electrocardiogram demonstrated sinus rhythm with new-onset ST depression in anterior leads, T-wave inversion in inferolateral leads, 1st degree A-V block, biatrial enlargement, left axis deviation, and ST elevation, findings concerning for possible myocardial injury (see Figure 1). Bedside echocardiography was performed at a heart rate of 115 beats/minute demonstrating hyperdynamic biventricular function without evidence of pericardial effusion or regional wall motion abnormalities and normal biventricular systolic and diastolic function. Pulse-wave Doppler interrogation of the LVOT in the apical long axis view demonstrated a velocity-time integral (VTI) of 14.7 cm. Using the LVOT VTI method to measure cardiac output (LVOT area × LVOT VTI), the stroke volume (SV) was 42 mL. The body surface area (BSA) of the patient was 1.14 m^2^, thus the cardiac output (SV × HR) was 4.83 L/min and cardiac index (cardiac output/BSA) was 4.24 L/min/m^2^. Laboratory investigation revealed hyperosmolar anion gap metabolic lactic acidosis (anion gap 28, pH 7.05, bicarbonate 11 mmol/L, lactate 16 mmol/L, and osmol gap 24). Distal tissue perfusion appeared adequate secondary to ScvO2 90% and CO2 gap 1 (central PvCO_2_-PaCO_2_). Differential diagnoses of septic shock, PG toxicity, malignant hyperthermia, and adrenal insufficiency were considered. Subsequent therapy included broad spectrum antibiotics, cessation of PG-containing medications (pentobarbital), cessation of volatile anesthetic, administration of stress-dose hydrocortisone, and initiation of intermittent hemodialysis. Acidosis and hemodynamic instability rapidly normalized after the institution of hemodialysis and the electrocardiogram findings normalized. Blood cultures remained negative. A random cortisol level 16 hours prior to the onset of shock was 5.8 mcg/dL. Renal impairment occurred following the episode of acute decompensation on hospital day 3 before initiation of hemodialysis (peak creatinine 1.9 mg/dL) and quickly resolved (creatinine 1.1 mg/dL following the first hemodialysis treatment). The diagnosis of PG toxicity was confirmed when the PG level on predialysis serum sample returned in the toxic range at 35 mg/dL.

## 3. Discussion

We present a case of acute, refractory, distributive shock with hyperosmolar anion gap metabolic lactic acidosis secondary to PG toxicity in a patient receiving PG at doses previously believed to be associated with low risk of toxicity. While toxicity has been reported to occur at PG serum levels above 18–25 mg/dL [3, 4, 7, 8], osmol gap has been suggested as a more useful surrogate measurement given the relative speed at which an osmol gap can be obtained relative to PG levels [9, 10]. In pediatric patients receiving continuous lorazepam infusions, a guideline for monitoring the osmol gap was recently developed with the recommendation to switch to an alternative sedative if the osmol gap is ≥12 mOsm/kg [10]. Since 30% of PG is excreted via the kidneys as a glucuronide conjugate and the remainder excreted unchanged in the urine or metabolized to intermediary byproducts (lactate, CO_2_), renal impairment is a known risk factor for the development of clinical toxicity [1]. While this patient did develop renal dysfunction during the episode of shock, we hypothesize that the etiology of his renal impairment was multifactorial secondary to both hypoperfusion and PG toxicity. While PG toxicity likely caused the hemodynamic collapse, PG has also been shown to be directly cytotoxic to the proximal renal tubular cells [11–13]. As dialysis will remove both PG and creatinine, the exact etiology of his renal dysfunction is unknown. While clinical presentation of PG toxicity may mimic septic shock, all tests for sepsis were negative, and our patient abruptly improved in response to therapies directed at PG toxicity, namely, dialysis. This is a unique case of PG toxicity because this patient received therapy within currently accepted medication dosing practice, and his average total daily exposure of PG was at or below 1 g/kg/day. Furthermore, to our knowledge, this is the first case in pediatrics documenting hemodynamic physiologic measurements by echocardiography in an adolescent with clinical manifestations of PG toxicity.

In the United States, there are no formal dosing recommendations of PG as an excipient in medications from the FDA. The European Medicines Agency (EMA) published a 2003 guideline regarding labeling of excipients of medicinal products for human use. They recognized that certain excipients, including PG, are inert at low doses but may pose a risk to humans at higher doses. The guideline mandated that a warning statement be included in the PG package labeling which was to include a “threshold dose” of 200 mg/kg/day in children [14]. This threshold dose was defined as the dose at which a pharmacologic effect might be expected, but it is not the highest acceptable daily dose and thus not dosing limit. In a 2014 draft revision of the guideline, the threshold dose in children was increased to 500 mg/kg/day following a review of the published safety data for PG [15]. Our experience demonstrates that abiding by the currently extrapolated pediatric limit (1 g/kg/day) published in the extant literature is insufficient to prevent the development of this toxidrome in critically ill children. Given that there are insufficient data both to affirm the safety of the lower dose suggested by the EMA and to provide formal recommendations from the FDA, we suggest that practitioners should be knowledgeable of these deficits and remain vigilant in regard to assessing for PG toxicity, even at doses believed to be safe.

## 4. Conclusion

In conclusion, it is imperative that health care providers maintain a high index of suspicion for PG toxicity while treating patients receiving PG-containing medications and consider surveillance osmol gap monitoring for prevention and early intervention of clinical toxicity, particularly in the face of hyperosmolar anion gap metabolic lactic acidosis with electrocardiogram findings consistent with ischemia.

## Abbreviations

PG: Propylene glycol
FDA: United States Food and Drug Administration
LVOT: Left ventricular outflow tract
VTI: Velocity-time integral
SV: Stroke volume
BSA: Body surface area.
